# Management and Treatment of Grover’s Disease: A Case Report and Review of Literature

**DOI:** 10.7759/cureus.24082

**Published:** 2022-04-12

**Authors:** John M Sousou, James M Fritsche, Brandon R Fernandez, Mahesh R Tummala, Randy Scott

**Affiliations:** 1 Family Medicine, Lake Erie College of Osteopathic Medicine, Jacksonville, USA

**Keywords:** skin biopsy, dermatitis, topical steroids, papulovesicular rash, intraepidermal acantholysis, grover disease

## Abstract

This case report details a rare case of Grover's disease in an 80-year-old Caucasian male complaining of a rash across his chest over the last three to four months. The patient has a past medical history of essential hypertension, hyperlipidemia, osteoarthritis of the knee, chronic gastroesophageal reflux disease (GERD), supraventricular tachycardia, status post prostate cancer, and restless legs syndrome. During his initial evaluation, he was found to have a pruritic, erythematous, papular rash most notably along his upper trunk and chest. The patient utilized multiple lotions, emollients, and anti-itch creams with minimal relief of his symptoms and presentation. Following a referral to Dermatology, a biopsy of the rash was conducted, which revealed intraepidermal acantholysis, the hallmark finding for a diagnosis of Grover's disease. Subsequently, he was treated with a topical triamcinolone acetonide 0.1% cream for 14 days. This study details a case of Grover's disease along with potential comorbidities and contributing factors in order to further understand the pathogenesis and etiology of this relatively rare condition.

## Introduction

Transient acantholytic dermatosis, commonly known as Grover's disease, is an acquired yet idiopathic erythematous papulovesicular rash often found on the central chest [1]. Grover disease has been diagnosed in roughly 0.1% of the total population, with a male-to-female ratio of 2.4:1, and an average onset at 61 years of age [2]. This condition primarily presents as a moderately pruritic, erythematous, papulovesicular rash most commonly located on the trunk, notably on the chest and back. Although the etiology and pathogenesis of this condition are not well known due to its rarity, the course of this disease is typically benign. A diagnosis of Grover's disease may be made clinically; however, there are a few notable tests to confirm the diagnosis and rule out other similar dermatological conditions, such as pemphigus vulgaris. Direct immunofluorescence staining and a potassium hydroxide (KOH) prep test will be classically negative for Grover's disease. The definitive diagnosis is made through a skin biopsy, which reveals intraepidermal acantholysis, indicating a loss of adhesion between keratinocytes within the epidermis. 

A data extraction analysis, conducted by Gantz et al. in 2017 [3], on the demographics associated with 69 cases of Grover's disease revealed that the majority of patients were male (71%) [3]. In addition, of the 35 cases for which the ethnicity of the patient was provided, 74% of patients were Caucasian. Previous research on Grover's disease has revealed the possibility of exacerbating factors contributing to the course of this rash, including heat, sweating, renal failure, and a history of malignancy [2]. In a study on potential comorbidities associated with Grover's disease, the majority of cases (61%) were linked to a previous history of malignancy, with 32% of those cancers being nonhematologic [3]. Further, of the total number of patients with a history of malignancy, 62% of them had received chemotherapy. Interestingly, an increased frequency of Grover's disease has also been found in association with patients who are bedridden or hospitalized for an extended period of time. In a study of 72 patients diagnosed with Grover disease, 21% were found to be bedridden [4].

Grover's disease often resolves spontaneously within a year; hence, management is centered around symptomatic relief and disease prevention [5]. Pruritus symptoms are treated with topical corticosteroids and antihistamines [1]. For more refractory cases, options like phototherapy, retinoids, and systemic corticosteroids can bring on symptomatic relief [5]. Due to the rarity of Grover's disease and lack of recent progress in treatment options, this case shines light on patients with this condition and management of symptoms.

## Case presentation

An 80-year-old male presented to the primary care office for an annual wellness visit and chief complaint of a new-onset rash across his chest. Past medical history includes essential hypertension, hyperlipidemia, osteoarthritis of the knee, chronic gastroesophageal reflux disease (GERD), supraventricular tachycardia, status post prostate cancer, and restless legs syndrome. Current medications include diltiazem 120 mg bid, lisinopril 40 mg qd, omeprazole 40 mg qd, meloxicam 15 mg qd, atorvastatin 40 mg qd, myrbetriq 50 mg qd, oxybutynin 10 mg qd, aspirin 81 mg qd, and magnesium 250 mg qd. Recent lab results of CBC with differential, Iron panel, PSA, CMP14+eGFR, and lipid panel were all unremarkable. The patient has no history of food, medication, or environmental allergies.

**Figure 1:**
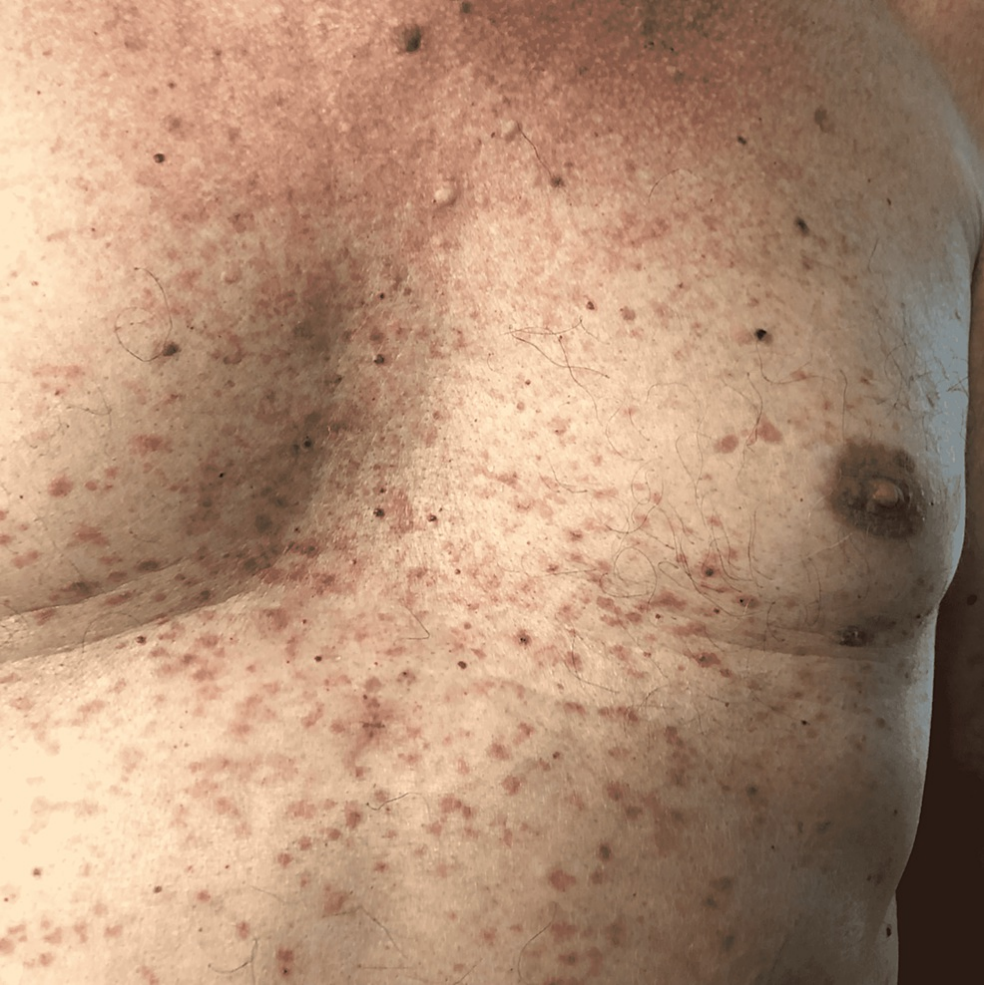
Photograph of the current patient’s chest, showing the characteristic erythematous, papular eruptions

The patient's only complaint since his previous visit one year ago is the development of a pruritic, erythematous, papular eruption across his entire chest that he has noticed over the last three to four months (Figure 1). The primary symptom reported by the patient is intense itching several times each day. The patient denies any pain, bleeding, or blistering associated with this rash. The patient has tried multiple lotions, emollients, and anti-itch creams with minimal relief. A referral was made to Dermatology for a biopsy of the lesion. Biopsy results of the lesions 10 days later revealed intraepidermal acantholysis; additionally, direct immunofluorescence staining was negative, ultimately indicating a diagnosis of Grover's disease. Upon his return to the clinic two weeks later, the biopsy results were reviewed with him, and triamcinolone acetonide 0.1% cream was prescribed; he was counseled to apply to the affected areas twice a day for 14 days. The patient was also instructed to avoid known triggers that may worsen his condition, such as heat, sweating, exposure to sunlight, prolonged bed rest, and dry skin.

The patient was scheduled for a follow-up visit in two weeks to determine if the prescribed triamcinolone acetonide 0.1% cream was a sufficient treatment for the management of his symptoms. In the follow-up visit two weeks post-treatment, the patient stated his symptoms have been properly controlled with minimal itching since he began using the prescribed steroid cream. Examination of his chest revealed persistence of the rash, however, decreased eruptions overall and no spreading. The patient was instructed that he may switch to over-the-counter (OTC) topical hydrocortisone cream as pruritic symptoms persist.

## Discussion

This case report highlights the presentation for Grover's disease, wherein a patient may present with a seemingly refractory dermatitis that is notably distinct in its papulovesicular morphology. Importantly, a history of malignancy along with the classic, yet nonspecific, pruritic, and erythematous rash should be considered for biopsy to confirm the diagnosis [3]. Although the condition can be exacerbated by sweating and can present concurrently with miliaria, which would be distinguished by its nodularity, it most frequently presents in the winter months [6]. This condition primarily presents as a moderately pruritic, erythematous, papulovesicular rash most commonly located on the chest, back, and upper arms. Although the etiology and pathogenesis of this condition are not well known due to its rarity, the course of this disease is typically benign.

As previously discussed, the pathogenesis and etiology of Grover's disease are still not well understood. Several studies have found various potential comorbidities and exacerbating factors strongly linked to this condition, including a history of malignancy. Our patient in the current study has a past medical history of prostate cancer. It is unknown whether or not his history of malignancy contributed to the development of Grover's disease, however as compared to previous research, there is a strong likelihood of this association. Additionally, several exacerbating factors have been proven to worsen the overall condition of Grover's disease. An example is prolonged exposure to sunlight causing increased heat and sweating, which are two direct factors that may worsen symptoms. Studies have also shown that prolonged bed rest may be an additional exacerbating factor in a patient with Grover's disease [4]. Our patient was instructed to avoid each of these triggers, as his symptoms persist, due to the negative impact they may have on his overall presentation and outcome.

Management of the patient’s treatment with topical steroids was an effective primary treatment for this condition due to alleviation of symptoms, decreased risk of adverse side effects, and cost-effectiveness [7]. Although some cases of Grover's disease resolve spontaneously, it is important to provide symptomatic relief, especially in patients with other comorbidities. Since there is no known cure for Grover's disease, the primary goal of treatment is centered around management of the patient’s symptoms as they persist. Although Grover's disease can be diagnosed clinically, it is imperative to rule out other potential diagnoses with similar presentations. Additional research is needed to determine the link between Grover's disease and malignancy, especially in the absence of other autoimmune conditions, which can otherwise explain the cause of acantholysis.

## Conclusions

Grover's disease is a rare dermatological condition that is currently not well understood and its etiopathogenesis still remains unclear. Several studies have shown potential comorbidities, contributing risks, and exacerbating factors linked to the development of this erythematous papulovesicular rash. This condition is typically a benign and self-resolving disease that can be diagnosed through a biopsy of the lesions, which reveals the hallmark finding of intraepidermal acantholysis. Due to the rarity of the disease, no definitive treatment is currently established, however topical steroids such as triamcinolone acetonide 0.1% cream have been proven to be successful for symptomatic treatment. More studies however are needed to further determine the etiology and pathogenesis of Grover's disease, which would aid our understanding of potential associations with this condition and the proper management throughout its course.
